# *In vivo* noninvasive microscopy of human leucocytes

**DOI:** 10.1038/s41598-017-13555-1

**Published:** 2017-10-12

**Authors:** Matan M. Winer, Adel Zeidan, Daniella Yeheskely-Hayon, Lior Golan, Limor Minai, Eldad J. Dann, Dvir Yelin

**Affiliations:** 1 Department of Biomedical Engineering, Technion-Israel institute of Technology, Haifa, Israel; 2 Department of Hematology and Bone Marrow Transplantation, Blood Bank and Aphaeresis unit, Rambam Medical Centre, and the Bruce Rappaport Faculty of Medicine, Technion, Haifa, Israel

## Abstract

Leucocytes play a key role in our immune system, protecting the body against infections using a wide range of biological mechanisms. Effective imaging and identification of leucocytes within the blood stream in patients is challenging, however, because of their low volume fraction in the blood, the high tissue scattering and the rapid blood flow. Spectrally encoded flow cytometry (SEFC) has recently been demonstrated effective for label-free high-resolution *in vivo* imaging of blood cells using an optical probe that does not require mechanical scanning. Here, we use SEFC to noninvasively image leucocytes at different imaging depths within small vessels in human volunteers, and identify visual differences in cell brightness and nuclei shapes, that would help distinguish between the two most abundant leucocyte types. The observed differences match the *in vitro* characteristics of isolated granulocytes and mononuclear cells. The results prove the potential of the system for conducting differential leucocyte count and as an effective research tool for studying the function and distribution of leucocytes in humans.

## Introduction

Leucocyte (white blood cells) counting and classification constitute a major part of routine blood tests, providing strong indication of the status of the immune system. Differentiating between the various types of leucocytes is often required for the diagnosis of a variety of illnesses, including allergies^1^, various malignancies^2^ and immunodeficiency diseases such as the acquired immunodeficiency syndrome^3,4^. The most common types of leucocytes in healthy adults are the neutrophils (a type of granulocyte cells) and the lymphocytes (a type of mononuclear cells), which typically comprise approximately 62% and 30% of the total leucocyte population, respectively^5^. Differentiation between these two main cell types is often required for identifying the sources of infection in ill patients; higher-than-normal neutrophil count may indicate active immune response to bacterial or fungal infection, while high lymphocyte count may indicate viral infection^6^. An accurate differential neutrophil-lymphocyte count is crucial for reducing the prescription of unnecessary antibiotics, which is known as one of the main challenges of modern medicine^7^. As part of the complete blood count (CBC), all five leucocyte types (neutrophils, eosinophils, basophils, lymphocytes and monocytes) are counted and reported as a percentage and as an absolute number per volume^8^. Leucocyte abnormalities such as atypical cell size and nucleus-to-cytoplasm volume ratio could be visualized using microscopic examination of stained blood smears^9^ for the diagnosis of specific illnesses, including dysgranulopoiesis, acute myeloid leukemia and chronic granulocytic leukemia. Recently, leucocyte count at the point of care has become possible using compact instrumentation^10^ including microfluidic single-cell impedance^11^ and sheathless microchip^12^ flow cytometers. Unlike conventional flow cytometry that requires extraction of several milliliters of blood, these methods can measure blood parameters using only a few microliters extracted by a finger prick, involving less pain, lower risk of infection, and faster results. Clearly, noninvasive differential leucocyte count, i.e. identifying and counting these cells without any extraction of blood, would be beneficial in many applications, both scientific – owing to the ability to study leucocytes *in situ*, and clinical – due to lower risk of infection, less pain and real-time results^13^.

In animal models, where the use of fluorescence labeling is common^14^, leucocytes have been tracked and counted using slit detection and confocal imaging^14,15^, widefield microscopy^16,17^, acoustic focusing of flowing cells^18^ and photoacoustic flow cytometry^19,20^. Improved penetration depth has been demonstrated using near infrared light^21^ and two-photon excited fluorescence^22^. In contrast to animal experiments, however, human subjects cannot tolerate large doses of fluorophores required for selective leucocyte staining. Furthermore, leucocytes have relatively small volume fraction in the blood and hence negligible absorption and scattering of visible and near-infrared light, which prevent their detection by conventional diffused optical sensing techniques such as diffused spectroscopy and pulse oximetry. Recently, Wu *et al*. have demonstrated label-free leucocytes imaging in humans using laser scanning third harmonic generation (THG) microscopy at high frame rates that reduce motion artefacts^23^.

In this work, we employ a recently developed system for noninvasive imaging of blood cells, termed spectrally encoded flow cytometry (SEFC)^24,25^, for high-resolution microscopy of leucocytes within small blood vessels in human volunteers. By imaging at different depths below the vessel walls we study the leucocyte appearance and note two main cell types: one with bright dots and a dark multi-lobed region and one with relatively dim speckled appearance. Based on early *in vitro* imaging experiments^24^ we find strong resemblance between the leucocyte types observed *in vivo* and the SEFC images of granulocytes and mononuclear leucocytes. The results demonstrate the potential of SEFC to noninvasively count leucocytes and differentiate between their two main subtypes in humans.

## Methods

**Figure 1:**
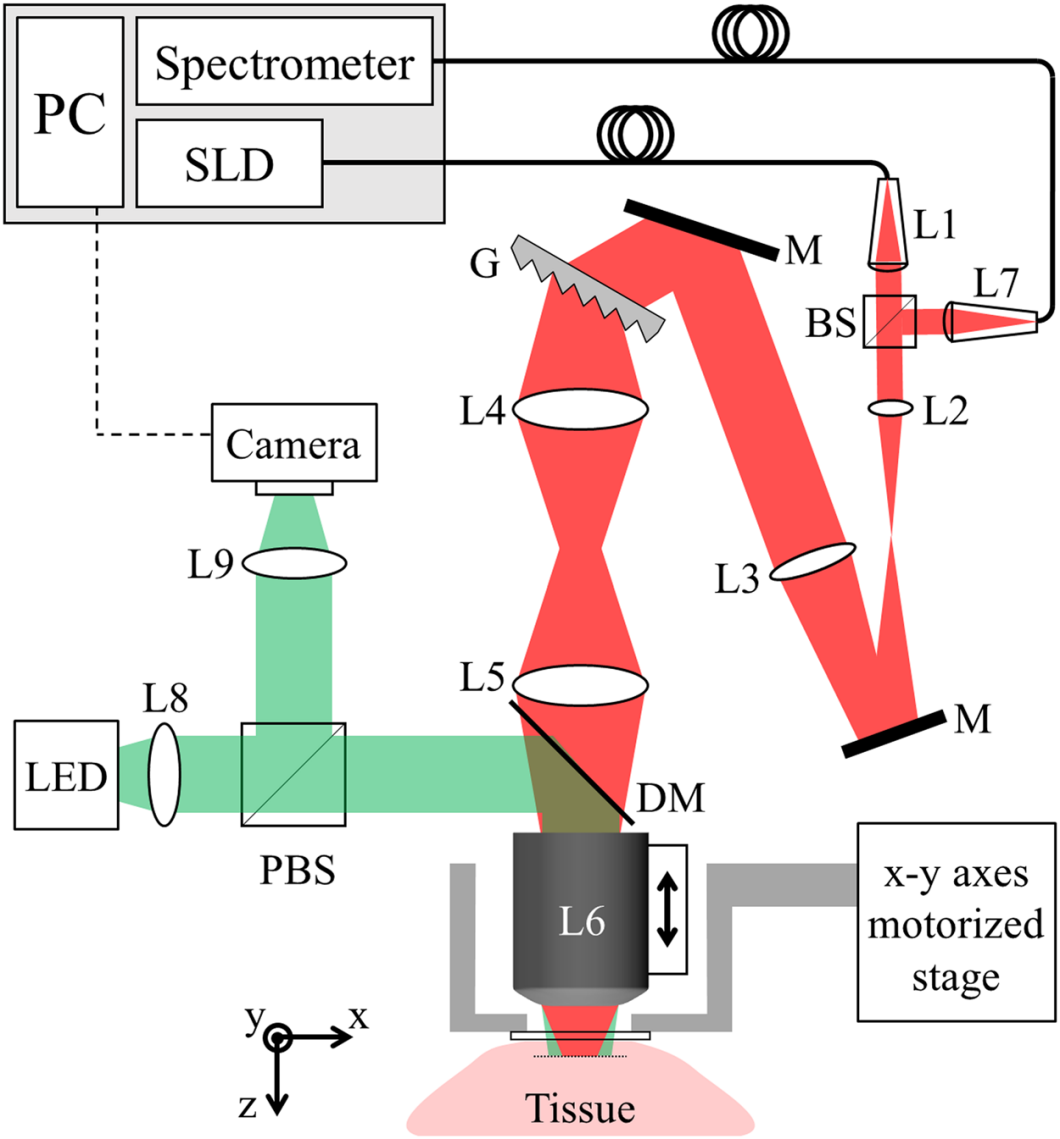
Schematic illustration of the *in vivo* SEFC system. L1-9: lenses. M: mirror. G: diffraction grating. BS: beam splitter. PBS: polarizing beam splitter. DM: dichroic mirror. SLD: super-luminescence diode.

The SEFC system used for this study (Fig. 1) is based on a system previously described in ref.^24^. Briefly, broadband light from a fiber-coupled super-luminescent diode (Superlum, 840 nm central wavelength, 50 nm bandwidth) was collimated using an aspheric lens (L1, 11 mm focal length) into a 2.4-mm-diameter beam, expanded by a 3× achromatic telescopic configuration (L2 and L3, 25 mm and 75 mm focal lengths, respectively), diffracted by a transmission grating (1200 lines/mm, Wasatch Photonics), and imaged onto the entrance aperture of a water-immersion objective lens (L6, 60×, 1.2NA) using a unit-magnification telescope (L4 and L5, 50 mm focal lengths). Light reflected from the tissue propagated back through the same optical path, deflected by a non-polarizing beam splitter (BS) and coupled using an 11-mm-focal length lens (L7) into a second single-mode fiber that provided the confocal pinhole of the system. The light from the fiber was analyzed by a home-built high-speed spectrometer with up to 70k spectra/s and 0.06 nm spectral resolution. The resulting spectrally encoded confocal transverse line spanned approximately 250 μm, with lateral (x-axis) and axial (z-axis) resolutions of approximately 0.7 μm and 1.5 μm, respectively. A widefield imaging channel for maneuvering the spectrally encoded line to a target blood vessel comprised of a light emitting diode (530 nm central wavelength, LED), an aspheric lens (15 mm focal length, L8), a polarizing cubic beam splitter (PBS) for reducing glare and surface reflections, an achromatic lens (50 mm focal length, L9) and a camera (IDS, 20 frames/s). A dichroic mirror (DM) was used to couple the widefield imaging channel into the optical path of the spectrally encoded imaging channel.

*In vivo* imaging was conducted within the inner lower lip of a healthy volunteer, where the thin epidermis of the oral mucosa allows imaging within vessels approximately 50 μm - 150 μm below the tissue surface. All methods were carried out in accordance with relevant guidelines and regulations, and were approved by the Rambam Medical Center Helsinki Committee (0207-11-RMB). After obtaining an informed consent, the volunteer was seated in front of the system, placing his chin on a chin-rest and positioning the inner lower lip against the cover glass that was attached to an aluminum cap. Under the visual guidance of the widefield imaging channel, the location of the spectrally encoded line was adjusted by laterally moving the aluminum cap (and hence the tissue in contact) with respect to the objective lens using a computer-controlled two-axis translation stage (M-122, Physics Instruments). Imaging depth was adjusted by moving the objective lens using a separate linear stage for reducing coupling between the three axes of motion. The system continuously acquired two-dimensional images (1024 lines × 2048 pixels per spectrally encoded line) at 5 kHz line acquisition rate (200 μs line exposure). Image data from both the confocal and widefield imaging channels were stored and displayed in real time using custom-built software (LabVIEW, NI).

## Results

**Figure 2:**
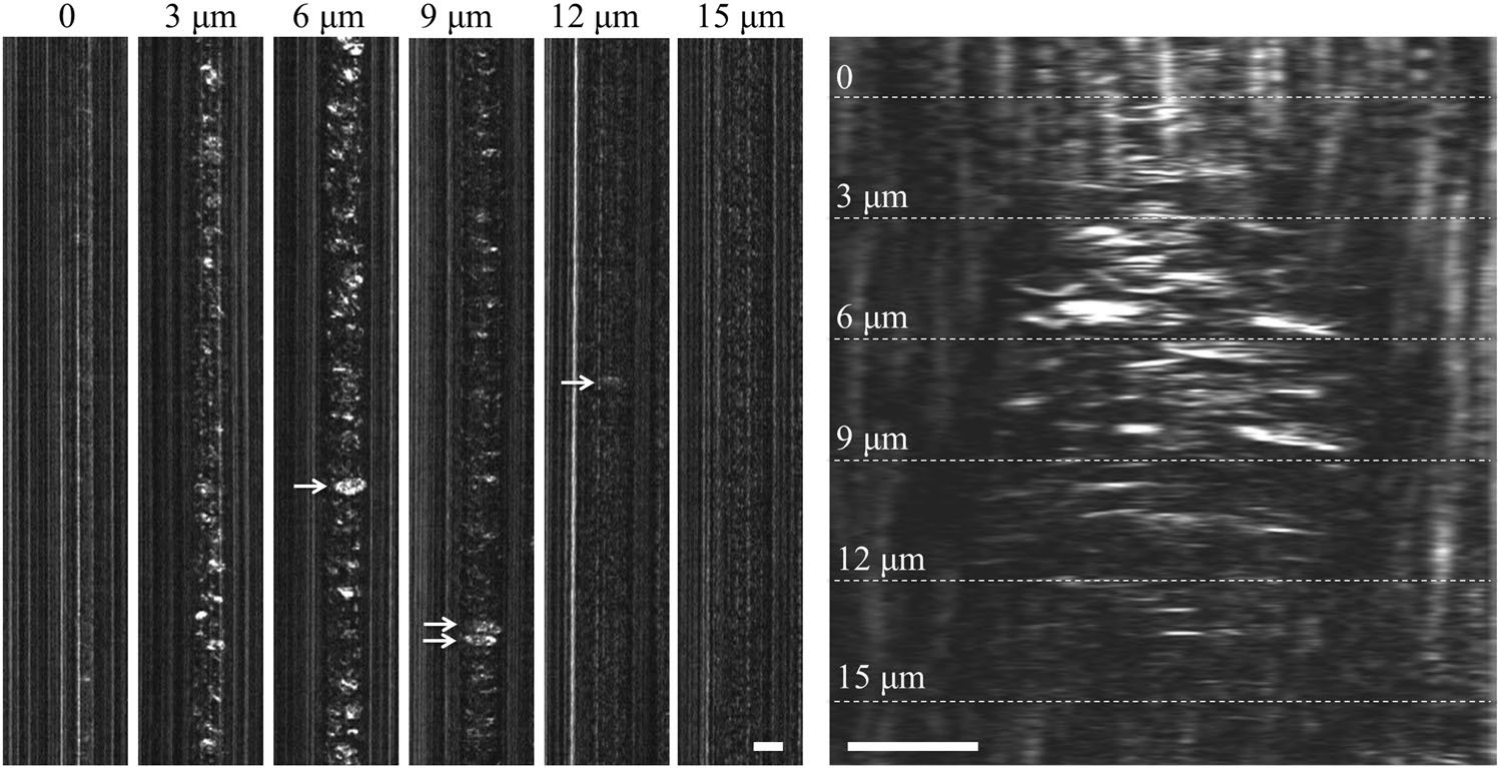
Left panels: Imaging at different depths in a small capillary vessel. Each panel corresponds to 80 ms of continuous acquisition. Arrows mark suspected leucocytes. Right panel: A constant z-axis scan of the spectrally encoded line across a small blood vessel of similar size. Dashed lines denote the approximate depths of imaging corresponding to the left panels. Scale bars represent 10 μm.

In order to study the SEFC imaging characteristics of leucocytes in small blood vessels, the spectrally encoded line was placed at different depths across a 15-μm-diameter capillary vessel (Fig. 2). When the confocal imaging plane overlapped with the front vessel wall (*z* = 0), only vertical lines were visible, resulting from reflections from the vessel wall. At 3 μm below the vessel wall erythrocytes (red blood cells) were visible in various shapes and patterns characterized by smooth elongated arcs and curves; residual reflections from the vessel wall were occasionally interfering with the erythrocyte reflections at this depth. The blood cells, including leucocytes (arrow), were clearly visible approximately 6 μm into the blood stream, where the confocal sectioning completely eliminated vessel wall reflections. At 9 μm below the front vessel wall erythrocyte brightness had decreased due to optical aberrations caused mainly by the shallower erythrocyte flow; the bright leucocytes (arrows), however, were still visible. Both erythrocytes and leucocytes appeared dim at imaging planes deeper than 9 μm, and were often overwhelmed by reflections from the back vessel wall. A continuous full axial scan across a vessel is shown at the rightmost panel in Fig. 2 with dashed lines denoting the approximate imaging planes shown in the left panels. Note the bright reflections from the red blood cells between 3 μm and 9 μm, and the rapid signal drop at deeper imaging planes. Jagged features on the vessel wall correspond to vibrational motion artefacts caused by the motorized axial translation stage.

**Figure 3:**
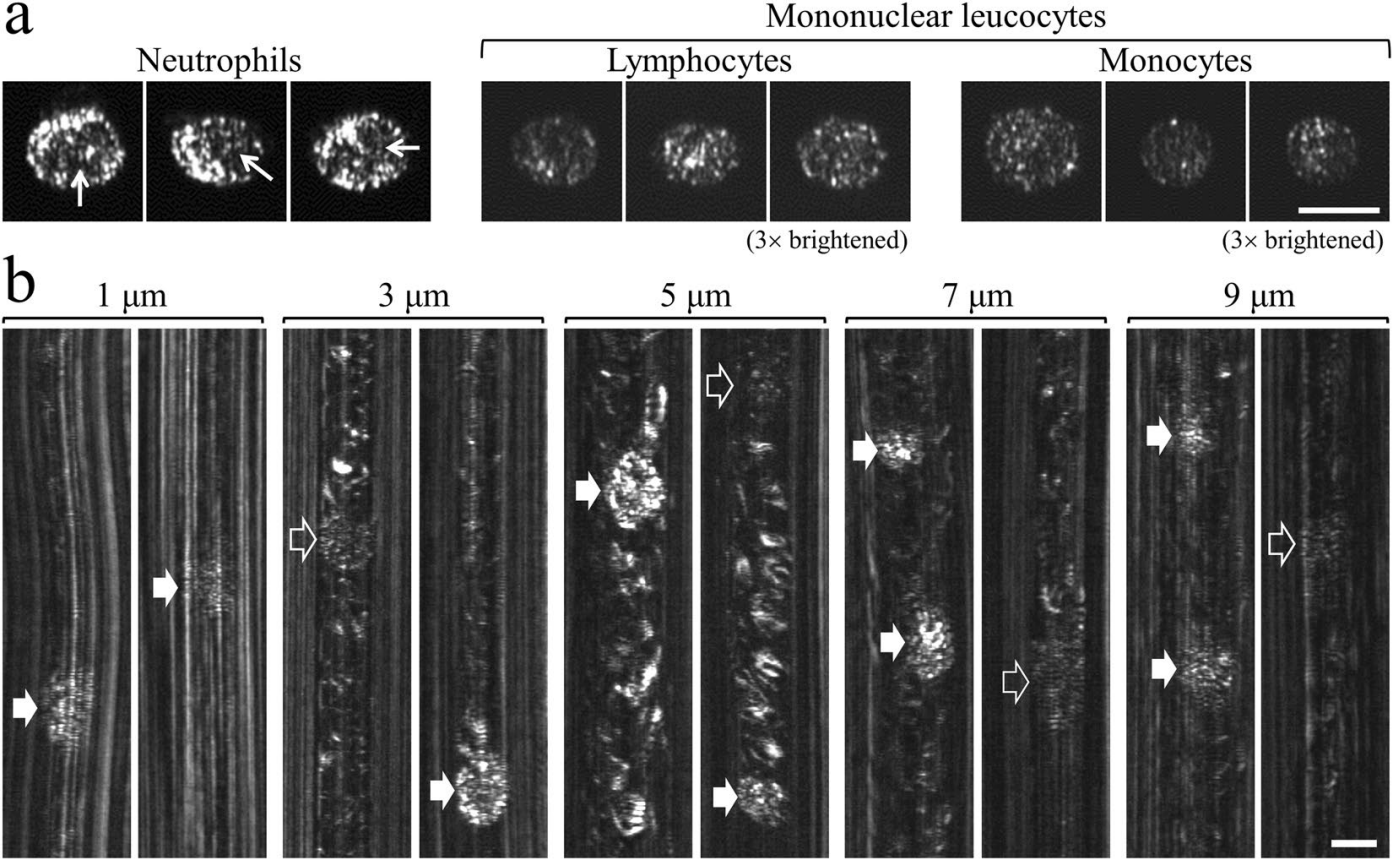
(**a**) Representative i*n vitro* SEFC images of individual neutrophils, lymphocytes and monocytes from sorted cell populations. Arrows mark multi-lobed nuclei. The lymphocyte and monocyte images were brightened by a factor of 3 to improve visibility. (**b**) Typical *in vivo* leucocytes images at different depths into a single 15-μm-diameter vessel. Filled and hollow arrows denote possible granulocytes and mononuclear leucocytes, respectively. Scale bars represent 10 μm.

In order to study the visual differences between the main leucocyte groups *in vivo*, we have first imaged (*in vitro*)^24^ homogeneous neutrophil, lymphocyte and monocyte populations that were sorted and isolated prior to imaging using immunodensity cell separation procedure for separating untouched cells directly from whole blood (RosetteSep™). Representative *in vitro* SEFC images of individual neutrophils (the most abundant granulocyte type), lymphocytes and monocytes revealed (Fig. 3a) that neutrophils were approximately 6-times brighter than mononuclear (lymphocytes and monocytes) cells, and were characterized by a noticeable multi-lobed dark nucleus (arrows in Fig. 3a). While the less abundant granulocytes eosinophils (typically a few percent in healthy adults) and basophils (less than 1%) were not imaged in this *in vitro* study, the granules in their cytoplasm would most likely result in a bright cytoplasm appearance similar to that of neutrophils. The three granulocyte types are thus expected to appear considerably brighter than the mononuclear leucocytes also *in vivo*, with apparent dark regions that correspond to their multi-lobed nuclei.

*In vivo* imaging was conducted on a 120-μm-deep, approximately 15-μm-diameter vessel at depths 1, 3, 5, 7 and 9 μm below the front vessel wall (Fig. 3b). At one micron below the vessel wall, the dominant wall reflections allowed only the brightest leucocytes, (filled arrows) to be visible. At 3 μm depth, bright leucocytes having similar appearance to neutrophils (bright granules, multi-lobed nucleus) were easily discerned from the red cells background. Leucocytes that resemble the *in vitro* imaged mononuclear lymphocytes and monocytes (Fig. 3a) were also visible (hollow arrows in Fig. 3b), characterized by dim speckled ovals lacking bright granules. Note that the relatively uniform and isotropic speckle patterns in leucocytes appear different from the elongated curves and arcs patterns of the erythrocyte reflections. At depth of approximately 5 μm below the vessel wall both the bright and dim leucocyte types were easily resolved on a bright erythrocytes background owing mainly to their unique speckled oval appearance. At this depth, the bright leucocytes were typically 3.2-times brighter than the dim leucocytes, and 1.2-times brighter than the erythrocyte background. At 7 μm and 9 μm imaging depths all leucocyte types became darker, yet could be easily resolved from the erythrocyte background. Evidently, the similarity in cell characteristics, i.e. bright cytoplasm granules and multi-lobed nucleus, between the bright leucocytes imaged *in vivo* and the neutrophils imaged *in vitro* strongly suggests that the bright leucocytes are granulocytes (neutrophils, basophils or eosinophils). Similarly, the darker leucocytes observed *in vivo* appear similar in brightness and speckled appearance to mononuclear leucocytes, and therefore could be identified as lymphocytes or monocytes.

**Figure 4:**
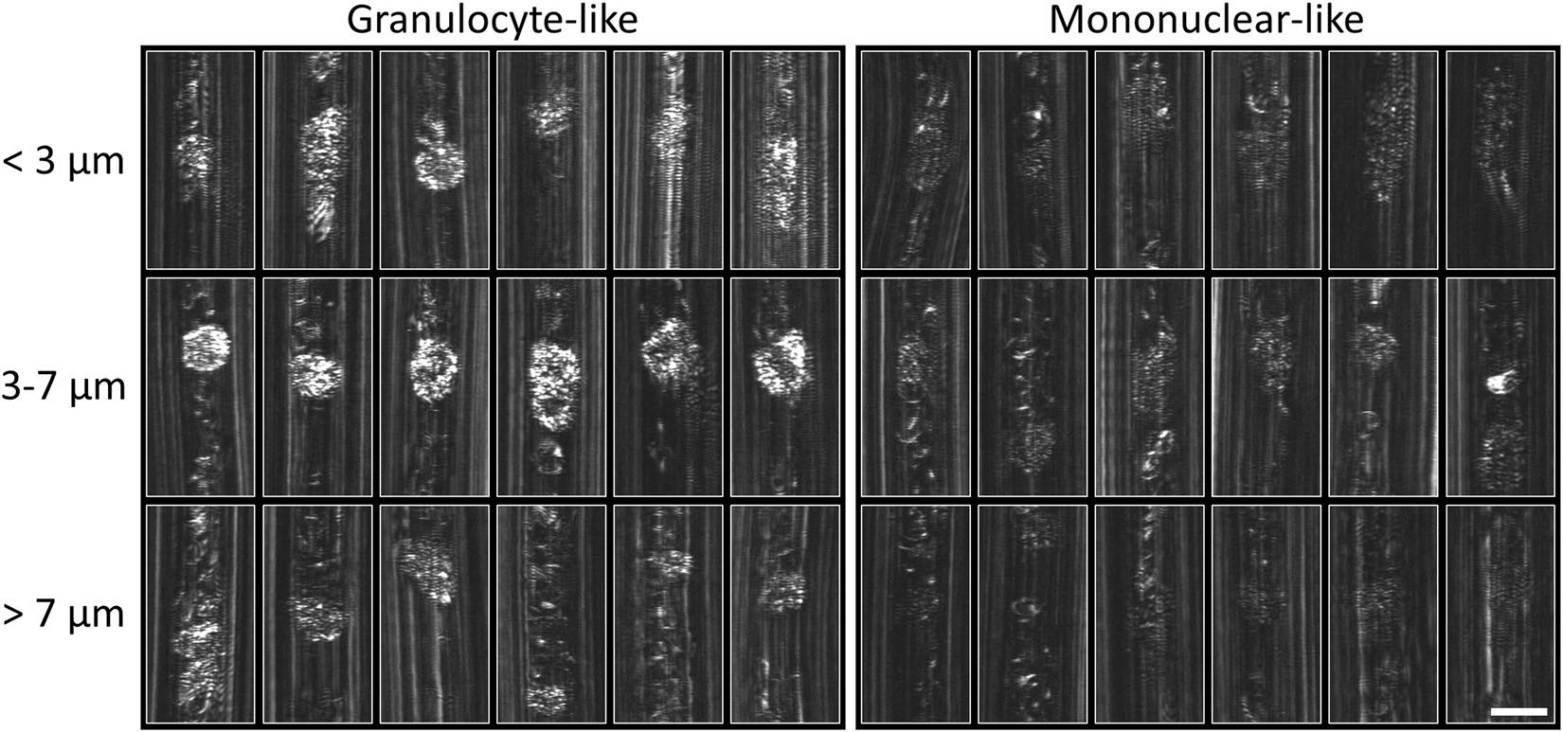
*In vivo* images of selected individual leucocytes grouped according to their apparent brightness and multi-lobed dark nucleus, and arranged in rows according to the imaging depth below the front vessel wall. Cytoplasm brightness difference and nucleus borders are most pronounced 3–7 μm below the vessel wall. Scale bar represents 10 μm.

**Figure 5:**
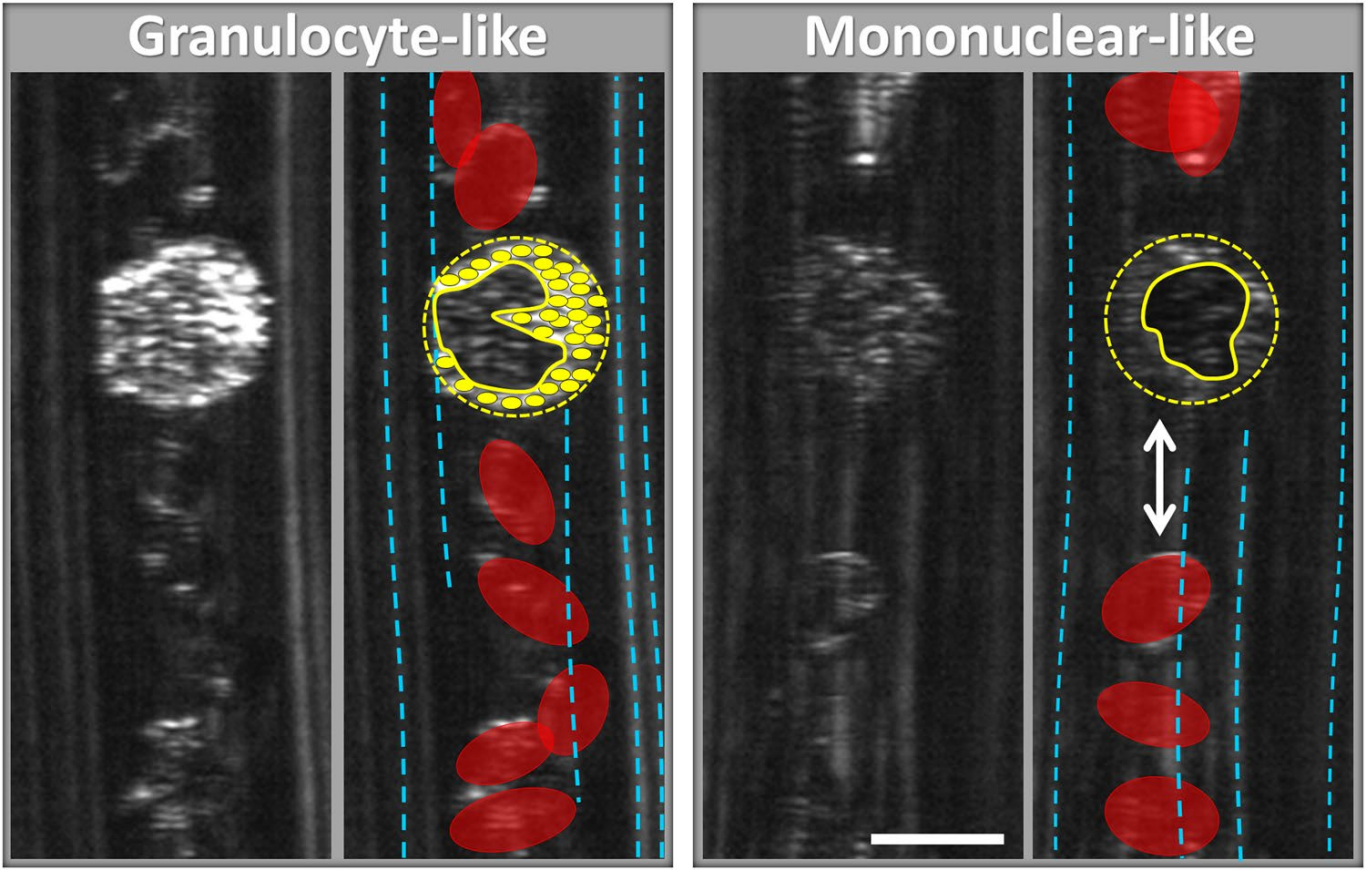
High-magnification images of leucocytes suspected as granulocyte (left) and mononuclear (right). Selected features in the images are highlighted in the right-hand side panels of each cell type, including cell boundaries (yellow circles), nucleus (black curve), granules (small yellow filled ovals), erythrocytes (red ovals), plasma gap (white double-headed arrow) and static vessel wall features (dashed blue lines). Scale bar represents 10 μm.

*In vivo* images of individual leucocytes grouped according to their visual similarity to granulocytes (left) and mononuclear cells (right) are shown in Fig. 4, sorted by the imaging depths below the front vessel wall (z = 0). All leucocyte images in Fig. 4 were captured flowing through small capillary loops; some of the cells were imaged twice while crossing the spectrally encoded line in both flow directions. At 3–7 μm below the vessel wall (middle row), the differences between the two groups are most significant – granulocyte-like cells are bright with dark nuclei and the mononuclear cells are dimmer than the brightest erythrocyte reflections but clearly noticeable as speckled ovals that are often separated from the erythrocyte background by the downstream plasma gap. Additional granulocyte-like and mononuclear-like leucocytes are shown in supplementary Figs. 1 and 2, respectively. High-magnification views of selected granulocyte-like and mononuclear-like cells are shown in Fig. 5 in the context of the surrounding blood vessel. An illustrated version of each image is presented on the right-hand panel of each leucocyte image. Multi-lobed dark regions (yellow curves) surrounded by small bright spots (filled yellow ovals) are visible in granulocyte-like leucocytes, most likely corresponding to a multi-lobed nuclei and highly scattering granule-rich cytoplasm, respectively. An additional feature evident in most leucocyte images is the erythrocyte-free plasma gap (double-headed arrow) that is formed downstream from most leucocytes due to their relatively large size and slower speed that disrupt the regular erythrocyte (red ovals) flow. These gaps, which appear darker compared to the erythrocyte background, help in leucocytes identification, particularly the dimmer leucocyte group, by increasing their local cell-to-background contrast. Dashed blue curves denote signals from static reflectors on the vessel walls.

**Figure 6:**
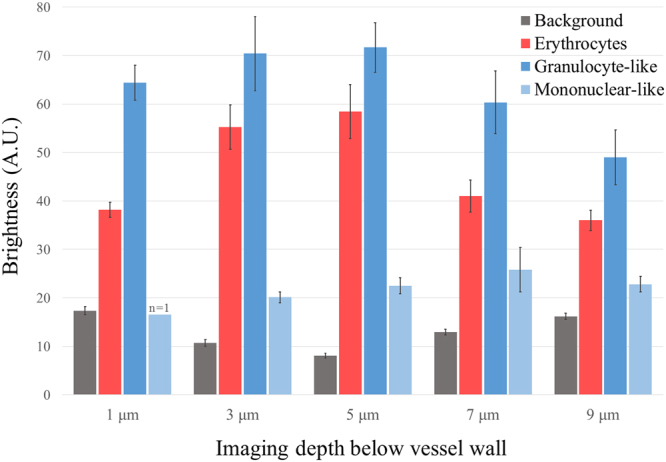
Mean brightness levels of leucocytes suspected as granulocytes and mononuclear at different depths below the front wall of a 15-μm-diameter vessel.

In order to define a single-parameter criterion for identifying and distinguishing granulocytes from mononuclear cells *in vivo*, the mean apparent brightness (average pixel values within manually segmented cell images) of eighty individual cells suspected as granulocytes (blue bars) and mononuclear cells (light blue bars) was calculated (ImageJ) and plotted in Fig. 6. The background levels of the red cells (red bars) and vessel walls reflections (grey bars) were calculated by averaging over pixel brightness at selected regions of dense erythrocyte population and cell-free regions, respectively. The resulting plot demonstrates the constant and significant brightness level difference between granulocyte-like and mononuclear-like cells. The mononuclear-like cells are consistently dimmer compared to the erythrocyte background, but brighter than the vessel background measured at the downstream plasma gap.

**Figure 7:**
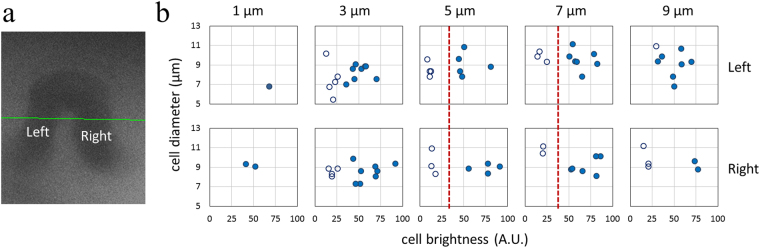
*In vivo* leucocyte differentiation in a capillary loop. (**a**) Widefield image of the capillary loop. Green line indicates the location of the spectrally encoded line. (**b**) Leucocyte brightness-size plot at different depths within the left and right-hand sides of the loop. Filled and hollow circles correspond to cells suspected as granulocytes and mononuclear, respectively.

In addition to mere brightness difference and multi-lobed nuclei, granulocytes are also known to be slightly larger on average than lymphocytes^6,24^ (but smaller than the less abundant monocytes). In SEFC, leucocyte diameter could be estimated by measuring their width along the spectrally encoded line (x axis), as the perpendicular time-encoded flow dimension is strongly dependent on flow velocity^26^. In order to identify single parameters for preliminary analysis of leucocytes, the spectrally encoded line was positioned across a capillary loop (Fig. 7a) during 288 seconds. Two-dimensional brightness-diameter scatter plots of 78 leucocytes are shown in Fig. 7b for different imaging depths. Most leucocytes in the loop were counted twice as they flew through the left (top panels) and right (bottom panels) capillary branches. By considering individual average cell brightness and the presence of a dark nucleus, the two main leucocyte populations were clearly noticeable at each imaging depth between 3 and 9 μm; 49 (62.8%) leucocytes were identified as granulocyte-like cells (filled circles) and 29 (37.2%) cells as mononuclear (hollow circles). Dashed red lines in the plots for 5 μm and 7 μm depths mark a potential decision-making threshold for differentiation between the leucocyte types. Evidently, while the average brightness between the two leucocyte groups was significant – approximately 3.5 times for 5–7 μm depth, the plots do not reveal significant size differences (vertical axes), possibly due to the thin confocal optical sectioning layer (approximately 2 μm) that prevented accurate diameter estimation of nearly spherical cell shapes.

## Discussion

Previous results from our *in vitro* SEFC system^24^ using well-sorted leucocyte populations have shown that neutrophils (granulocyte cells) appear considerably brighter compared to mononuclear (lymphocytes and monocytes) cells and include a characteristic multi-lobed dark nucleus surrounded by a bright granule-rich cytoplasm. Because the cells were kept in near physiological conditions (autologous blood plasma diluted with phosphate buffered saline^24^), and as reflectance confocal microscopy is sensitive only to refractive index variations within the cells, it is thus expected that neutrophils will have similar appearance in both *in vitro* and *in vivo* images. The highly scattering granules are also found in eosinophils and basophils, the two additional members of the granulocyte family; hence all granulocytes are expected to have similar appearance in the SEFC images, i.e. multi- or bi-lobed nuclei and bright cytoplasm. Similarly, the dim speckled appearance of mononuclear cells in the *in vitro* experiments is expected to be maintained *in vivo*. The clear division between the two leucocyte groups in our *in vivo* images presented in this work, both in terms of cell brightness and dark nuclei, strongly suggests that these groups could be identified as granulocytes and mononuclear cells. This identification is particularly significant when imaging the cells at a selected depth range of 5–7 μm within small vessels. The granulocyte-mononuclear cells percentage of 62.8% and 37.2% measured *in vivo* is also in agreement with the percentage in healthy adults (typically 65% and 35%, respectively). Different leucocyte brightness levels in human were also observed by Wu *et al*. using THG microscopy^23^. THG microscopy allows some important benefits for noninvasive imaging, including high penetration depth and unique nonlinear contrast mechanisms. In comparison, SEFC allows higher imaging rates, scan-free optical system, lower peak and average illumination intensities, and scattering-based contrast mechanism.

Effective differential count of granulocytes and mononuclear leucocytes requires imaging at specific conditions that permit equal-probability identification of both cell types. We show that such conditions could be fulfilled within small vessels (10–20 μm diameter) at an imaging depth range between 5 μm and 7 μm into the vessel; at these depths all leucocytes flowing through the spectrally encoded line could be identified and counted, allowing for a potentially reliable estimation of their relative numbers. Furthermore, as the blood volume imaged by our system is known (based on the known focal depth and estimated flow velocity^26^), the absolute concentration of granulocyte and mononuclear leucocytes in the blood could be measured. Future clinical experiments with a large number of patients would determine the true correlation between SEFC differential count (relative and absolute concentrations) and the differential count obtained via the CBC. Imaging conditions that reveal granulocytes but do not permit detection of the dim mononuclear cells (shallow imaging depth, larger vessels, etc.) could be useful for improving the accuracy of the differential count by combining the relative granulocyte-mononuclear measurement with a more statistically significant granulocyte count.

Some challenges remain before the SEFC system could be useful for routine clinical studies in patients. First, a user-friendly interface that provides control over the image acquisition parameters and allows maneuvering of the confocal line across the tissue must be developed and implemented. Using lower-NA free-space objective lenses may improve penetration depth and reduce complexity. We are currently developing a soft silicone fixture that stabilizes the lip against the cover glass and enables identification and scanning of target vessels with less patient discomfort. Second, effective data processing algorithms for differentiating between the different leucocyte types based on their morphological features need to be developed and tested. Future software could incorporate pattern recognition codes and smart, adaptive learning algorithms for automated multi-parameter classification and analysis of large numbers of cells. Third, better confidence in the identification of the different leucocyte types could be gained using animal experiments with specific fluorescence labeling of each leucocyte type. Potential issues that would limit the validity of such approach may include the different physiology between humans and the animal model, as well as the unknown effect of the fluorescent labels on the measured cells. Finally, as *in vivo* human studies cannot involve specific fluorescence labeling of individual cell types, only a comprehensive patient study that compares the results of SEFC analysis to the gold standard of the CBC, would allow relating the SEFC leucocyte images to the true type of the cell; such experiments would provide reliable training sets for leucocyte identification *in vivo*. Complete differentiation between all various granulocyte types (neutrophils, basophils and eosinophils) and between the mononuclear cells (lymphocytes and monocytes) would also require additional *in vitro* experiments for sorting, isolating and imaging the rarest cell types, and defining specific morphological features that would allow reliable *in vivo* identification.

In summary, we have imaged individual leucocytes flowing within small blood vessels in the lip of a healthy human volunteer. By comparing *in vivo* images of individual leucocytes to *in vitro* images of sorted leucocyte populations, we have identified specific visual features that are unique to granulocytes and to mononuclear cells. Our results present a first preliminary analysis toward effective microscopy of leucocytes in patients for both scientific and clinical applications, providing an effective tool for studying the function and status of the immune system and its response against infections in real time.

## Electronic supplementary material

Supplementary Figures
