# Supplementary material for: In vivo noninvasive microscopy of human leucocytes
Source: Sci Rep. 2017 Oct 12;7:13031. doi: 10.1038/s41598-017-13555-1 (PMC5638923; doi:10.1038/s41598-017-13555-1)
Supplement: Supplementary file 1 — Supplementary Figures [file 41598_2017_13555_MOESM1_ESM.doc]

**In vivo noninvasive microscopy of human leucocytes**

Matan M Winer, Adel Zeidan, Daniella Yeheskely-Hayon, Lior Golan, Limor Minai, Eldad J Dann,^1^ and Dvir Yelin

*Department of Biomedical Engineering, Technion-Israel institute of Technology, Haifa, Israel*

*^1^ Department of Hematology and Bone Marrow Transplantation, Blood Bank and Aphaeresis unit, Rambam Medical Centre, and the Bruce Rappaport Faculty of Medicine, Technion, Haifa, Israel*

Supplementary figures

.


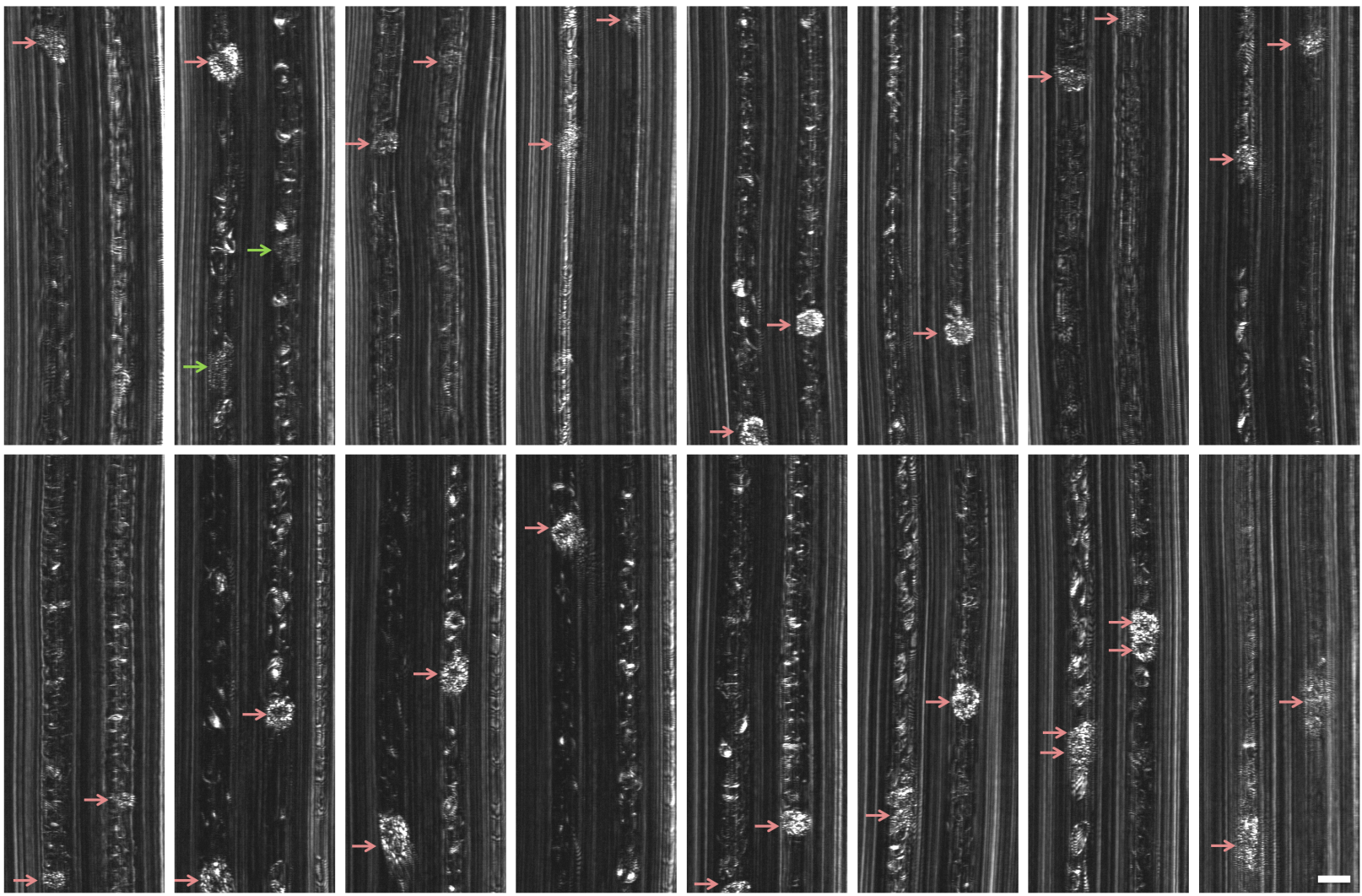


**Supplementary Figure 1:** Selected *in vivo* SEFC images of blood flow in a capillary loop showing leucocytes having visual similarities to granulocytes *in* *vitro* images. Red arrows mark the granulocyte-like cells. Green arrows mark mononuclear-like cells. Note that the capillary loop allows imaging each cell twice during each passage across the spectrally encoded line. The vertical separation between each cell image corresponds to the time between each appearance. Scale bar represents 10 μm.


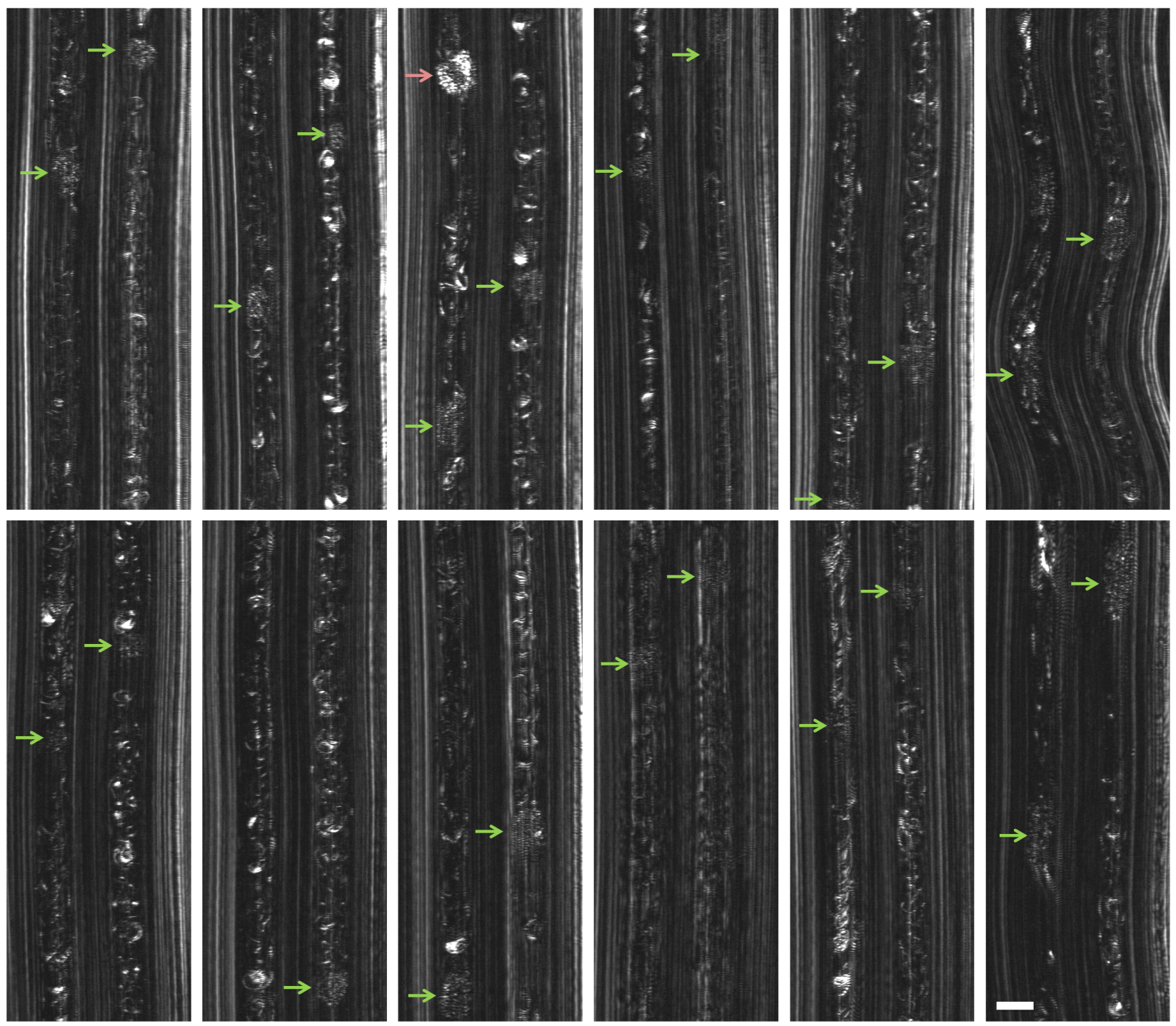


**Supplementary Figure 2**: Selected *in vivo* SEFC images of blood flow in a capillary loop showing leucocytes having visual similarities to mononuclear-cells *in* *vitro* images. Green arrows mark mononuclear-like cells. Red arrow marks a granulocyte-like cell. Note that the capillary loop allows imaging each cell twice during each passage across the spectrally encoded line. The vertical separation between each cell image corresponds to the time between each appearance. Scale bar represents 10 μm.
